# Zinc Influx Restricts Enterovirus D68 Replication

**DOI:** 10.3389/fmicb.2021.748546

**Published:** 2021-10-13

**Authors:** Shunan Liu, Xia Cao, Haoran Guo, Wei Wei

**Affiliations:** ^1^Department of Pharmacology, School of Pharmacy, Jilin University, Changchun, China; ^2^Key Laboratory of Organ Regeneration and Transplantation of the Ministry of Education, Institute of Translational Medicine, Institute of Virology and AIDS Research, The First Hospital of Jilin University, Changchun, China

**Keywords:** enterovirus, EV-D68, zinc influx, pyrrolidine dithiocarbamate, antiviral agents

## Abstract

Enterovirus D68 (EV-D68) is a respiratory viral pathogen that causes severe respiratory diseases and neurologic manifestations. Since the 2014 outbreak, EV-D68 has been reported to cause severe complications worldwide. However, there are currently no approved antiviral agents or vaccines for EV-D68. In this study, we found that zinc ions exerted substantial antiviral activity against EV-D68 infection *in vitro*. Zinc salt treatment potently suppressed EV-D68 RNA replication, protein synthesis, and infectious virion production and inhibited cytopathic effects without producing significant cytotoxicity at virucidal concentrations (EC_50_=0.033mM). Zinc chloride (ZnCl_2_) treatment moderately inhibited EV-D68 attachment. Time-dose analysis of EV-D68 structural protein VP1 synthesis showed stronger suppression of VP1 in the culture medium than that in the cell lysates. Furthermore, a zinc ionophore, pyrrolidine dithiocarbamate, which can transport zinc ions into cells, also enhanced the anti-EV-D68 activity of ZnCl_2_ treatment. Taken together, our results demonstrated that the enhancement of zinc influx could serve as a powerful strategy for the therapeutic treatment of EV-D68 infections.

## Introduction

Enterovirus D68 (EV-D68) of the *Enterovirus* genus (*Picornaviridae* family) was first isolated in 1962 (Schieble et al., 1967; D'Esposito and Postle, 2015). It has been found that EV-D68 particularly affects children, occasionally causing respiratory illness (Nafisa et al., 2021). It was initially considered a rare virus until the first large outbreak in 2014 (Midgley et al., 2014). Importantly, recent studies have found that EV-D68 infection not only triggers sporadic respiratory illness but is also associated with serious neurological conditions as acute flaccid myelitis (AFM), mainly occurs in pediatric patients (Midgley et al., 2015; Chang et al., 2017). Although EV-D68 is considered to be the main causative agent of AFM, therapeutic drug or treatment specifically for EV-D68 infections is still not available globally.

Enterovirus D68 is a non-enveloped, single-stranded, positive-sense RNA virus. Unlike most enteroviruses that encode ORF2p/uORF (Guo et al., 2019; Lulla et al., 2019), the EV-D68 genome has only one open reading frame (ORF). The ORF encodes a polyprotein precursor, which can be processed by viral proteases into four structural proteins (VP1, VP2, VP3, and VP4) and seven nonstructural proteins (2A, 2B, 2C, 3A, 3B, 3C, and 3D). The known EVD68 receptors are sialic acid and ICAM-5 (Liu et al., 2015a; Baggen et al., 2016; Wei et al., 2016). Thus far, the pathogenesis of EV-D68 infections remains poorly understood.

Zinc ion is the second most abundant transition metal ion in human bodies. It plays vital roles in many biological processes, including cell fate and development, gene transcription, and viral infection. Zinc ion inhibits viral replication by impairing polyprotein processing (Lanke et al., 2007), inhibiting transcription (Haraguchi et al., 1999; Fenstermacher and DeStefano, 2011), interfering with viral-cell membrane fusion to block viral binding and attachment (Suara and Crowe, 2004), and inhibiting viral genome replication (Korant et al., 1974; Bracha and Schlesinger, 1976). Furthermore, zinc plays a crucial role in enhancing host resistance to viral infections by sustaining immune homeostasis. Conversely, zinc deficiency impairs the cellular immune response and weakens cellular immunity. Compounds that are capable of transporting zinc into cells or redistributing cellular zinc exert a viral inhibitory effect.

In this study, we aimed to evaluate the inhibitory effects of intracellular zinc concentration on EV-D68 replication. We also aimed to enhance the antiviral activity of zinc salts using a zinc ionophore. Our results collectively provide proof-of-concept evidence for the clinical investigation of zinc supplementation in EV-D68 therapy.

## Materials and Methods

### Cells and Viruses

RD cells (CCL-136, ATCC, Manassas, VA, United States), A549 cells (CCL-185, ATCC), and HeLa cells (CCL-2, ATCC) were cultured in Dulbecco’s modified Eagle medium (DMEM; cat no: SH30022.01, HyClone, Logan, UT, United States) supplemented with 10% fetal bovine serum (FBS, REF: 04–001-1, Biological Industries, Beit HaEmek, Israel) and 1% penicillin/streptomycin solution. EV-D68 prototype Fermon (VR-1826, ATCC) and EV-D68 2014 US circulating strains, US/MO/14-18947(VR-1823D, ATCC) and US/KY/14-18953(VR-1825D, ATCC), were amplified in RD cells. A mixture of cell and supernatant was collected approximately 3days post-infection and subjected to multiple cycles of freezing and thawing. Then, the samples were clarified using low-speed centrifugation and passed through a 0.22-μm filter, and viral particles were pelleted through a 20% sucrose cushion in an SW28 rotor by centrifuging at 28,000rpm for 90min. The virus stocks were stored at −80°C until further use. All the experiments in this study were approved by the ethics committee of the First Hospital of Jilin University.

### Cell Viability Assay

The cells were treated with dose-gradient zinc when the confluency of RD cells was 80%. We prepared quadruplicate wells for each dose in a 96-well plate and filled each well with 100μl medium. A blank group was prepared without cells but filled with medium. Before adding zinc solution, the wells were washed off once with phosphate-buffered saline (PBS) to prevent the interference of dead cells. After 24h of incubation, the wells were washed off once with PBS and 10μl MTS (Biocompare, San Francisco, CA, United States) was added into each well, in dark conditions. Over the next 4h, the OD value was tested at 490nm by a 550 Bio-Rad plate reader every hour. The data were analyzed with GraphPad Prism 8.0 software (GraphPad Software, San Diego, CA, United States), and the IC_50_ was calculated.

### Virus Titer Assay

Viruses from the supernatants were isolated as previously described (Jiang et al., 2020). The supernatant was subjected to three freeze–thaw cycles. Then, the samples were centrifuged at 3,000rpm for 10min, and the supernatants were collected. Virus titers were determined based on the appearance of cytopathic effects (CPEs) in RD cells using a microtitration analysis in accordance with the Reed-Muench method (Reed and Muench, 1938). Virus titers were expressed as the 50% tissue culture infectious dose (TCID_50_).

### Viral RNA Quantification

Total RNA was extracted using a viral RNA isolation kit (Foregene, Chengdu, Sichuan, China) according to the manufacturer’s protocol. cDNA was generated using a reverse transcriptase kit (TransGen, Beijing, China). The viral RNA was quantified using qRT-PCR with the SYBR Green reaction mix (GenStar, Beijing, China) in a Roche LightCycler 480 (Roche, Basel, Switzerland). The following primer was used: *GAPDH* forward primer 5'-GCAAATTCCATGGCACCGT-3'; *GAPDH* reverse primer 5'-TCGCCCCACTTGATTTTGG-3'; EV-D68 forward primer 5'-TGTTCCCACGGTTGAAAACAA-3'; and EV-D68 reverse primer 5'-TGTCTAGCGTCTCATGGTTTTCAC-3'. The relative levels of EV-D68 RNA in different samples were determined using a comparative 2^−△△CT^ method and normalized to the *GAPDH* gene (Wei et al., 2016).

### Immunoblotting

Cells were harvested at various time points post-infection, washed twice with cold PBS, and lysed in lysis buffer [150mM Tris (pH 7.5) containing 150mM NaCl, 1% Triton X-100, and complete protease inhibitor cocktail (Roche)] and loading buffer [0.08M Tris (pH 6.8) containing 2.0% SDS, 10% glycerol, 0.1M DTT, and 0.2% bromophenol blue]. The solutions were boiled and vortexed for 10min and then centrifuged at 12,000rpm for 10min. Supernatant proteins were separated using SDS-PAGE and transferred to nitrocellulose membranes using a semi-dry apparatus (Bio-Rad, Hercules, CA, United States). The membranes were probed with primary antibodies [anti-EV-D68 VP1 polyclonal antibody (GTX132312, Genetex, Irvine, CA, United States) and anti-α-tubulin monoclonal antibody (A01410, GenScript, Nanjing, Jiangsu, China)] at 4°C overnight, followed by incubation with secondary antibodies (alkaline phosphatase-conjugated goat anti-rabbit IgG (code: 115-005-045, Jackson ImmunoResearch Laboratories, West Grove, PA, United States) and goat anti-mouse IgG (code: 115-055-062, Jackson ImmunoResearch Laboratories, West Grove, PA, United States), respectively for 1h at 25°C. The membranes were stained with 5-bromo-4-chloro-3-indolyl phosphate and nitrotetrazolium blue chloride (Sigma-Aldrich, St. Louis, MO, United States) and visualized for band quantification.

### Viral Attachment Assays

For virus attachment experiments, cells were first rinsed with cold PBS and then incubated with viruses (multiplicity of infection, MOI=0.1) at 4 or 37°C for 2h. Following this, the infected cells were washed with cold DMEM to remove unbound viruses. Total RNA was extracted using a viral RNA isolation kit (Foregene, Chengdu, Sichuan, China). The bound viral RNA was determined using qRT-PCR as described above.

### Statistical Analysis

All statistical analyses were performed using GraphPad Prism software 8.0 (GraphPad Software, San Diego, CA, United States). Differences among test groups were analyzed using ANOVA. A value of *p*<0.05 was considered statistically significant.

## Results

### Zinc Ions Inhibit Replication of EV-D68

**Figure 1:**
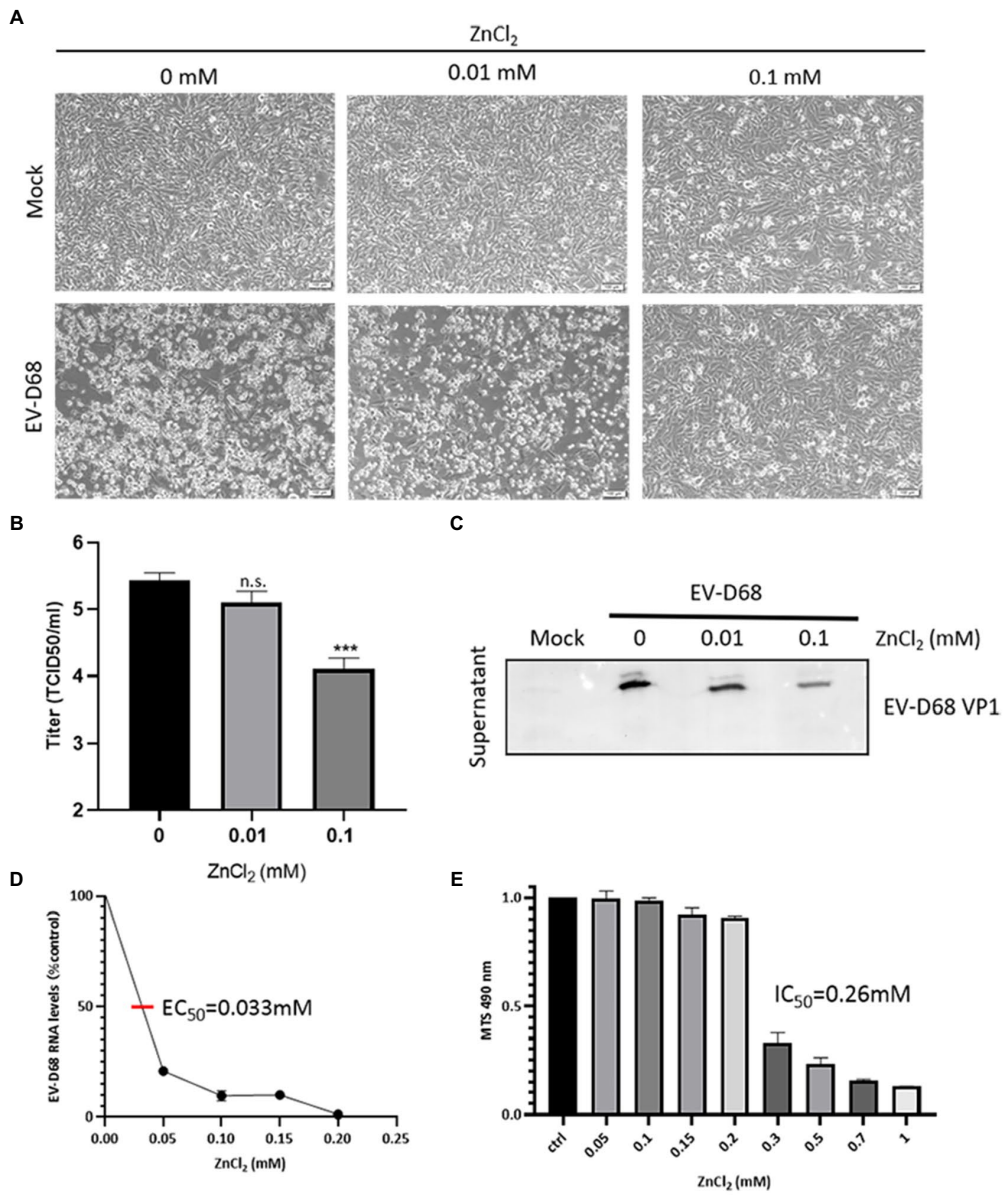
ZnCl_2_ inhibits enterovirus D68 (EV-D68) replication. **(A)** After a 2-h exposure to EV-D68, RD cells were treated with ZnCl_2_. The cells were incubated under standard conditions. Cytopathic effects (CPEs) were observed 48h post-infection (hpi). **(B)** Determination of progeny viral production. Supernatants were collected 48hpi, and viral titers were determined by standard plaque assay. Error bars indicate the SD (*p*<0.05, *p*<0.01, and *p*<0.001). **(C)** VP1 expression in the supernatant of ZnCl_2_-treated RD cells. The supernatant was collected at 48hpi, and the samples were prepared for western blot. **(D)** Effect of zinc on EV-D68 RNA replication (EC_50_=0.033mM). **(E)** Cell viability assay. Cellular toxicity of zinc chloride was evaluated by MTS assay.

To investigate the effects of zinc salts on EV-D68 infectivity, RD cells were treated with zinc chloride (ZnCl_2_), followed by challenge with an EV-D68 prototype Fermon (MOI=0.1). The CPEs were detected at 48h post-infection (hpi) in the control group, whereas ZnCl_2_-treated cells showed prominent resistance to CPEs (Figure 1A). The titers of total viruses were significantly suppressed upon ZnCl_2_ treatment (Figure 1B). Consistently, the viral structural protein VP1 in the supernatant of infected cells was also reduced (Figure 1C). The EC_50_ value of ZnCl_2_ against EV-D68 replication was approximately 0.033mM (Figure 1D). We also measured ZnCl_2_ cytotoxicity using the MTS assay and determined that the IC_50_ of ZnCl_2_ was 0.26mM (Figure 1E), which was much higher than the EC_50_ of the antiviral activity. The anti-EV-D68 activity of ZnCl_2_ was also observed in other permissive cells, namely A549 cells and HeLa cells (Supplementary Figure S1). Furthermore, we also confirmed the inhibitory effect of zinc sulfate and zinc acetate on EV-D68 replication (Table 1). Collectively, zinc ions could potently inhibit EV-D68.

**Table 1:** Effect of various zinc salts on EV-D68 replication.

Salt	Concentration	Virus titer	(mM)	(48hpi.)
Chloride	0	5.5
	0.01	5
	0.1	4
Sulfate	0	5
	0.01	4.5
	0.1	4
Acetate	0	5
	0.01	4.3
	0.1	4

*Zinc salts exert inhibitory effect on EV-D68 replication. The EV-D68-infected RD cells were treated with various zinc salts (zinc chloride, zinc sulfate, and zinc acetate). At 48hpi, the supernatant was collected for virus titer assay*.

### Zinc Ions Suppress EV-D68 Entry

**Figure 2:**
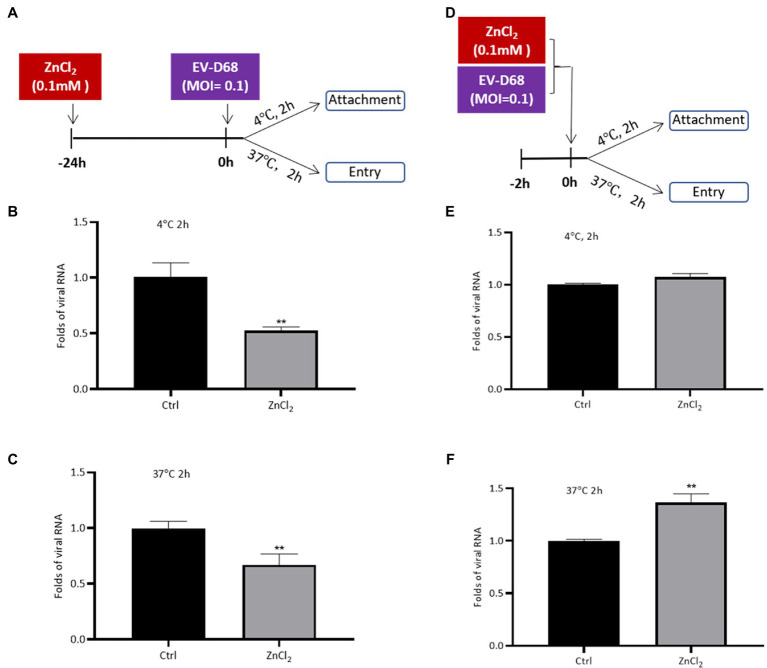
Zinc ions suppress EV-D68 entry. **(A–C)** RD cells pretreated with ZnCl_2_ for 24h. RD cells were precooled at 4°C for 30min before challenging with Fermon strain of EV-D68, and the cells were kept at 4 or 37°C for 2h. Unattached virus particles were washed off. The cells were collected for qRT-PCR assay to detect the viral replication. **(D–F)** EV-D68 was incubated with ZnCl_2_ for 2h at 37°C. The precooled RD cells were treated with this mixture at 4 or 37°C for 2h. The cells were collected at 2 hpi for qRT-PCR assay. Error bars indicate the standard deviation (*p*<0.05; *p*<0.01; and *p*<0.001).

We sought to determine the phase of the EV-D68 life cycle that was inhibited upon ZnCl_2_ treatment. ZnCl_2_-pretreated RD cells were incubated with equal amounts of EV-D68 particles at 4 and 37°C (Figure 2A). Two hours later, the unbound virions were washed off with cold PBS, and the attachment and entry of the viral RNA were determined using qRT-PCR. The results indicated that both virus attachment and entry were inhibited by ZnCl_2_ treatment (Figures 2B,C), compared to the control groups.

To eliminate the potential effects of the interaction between zinc ions and EV-D68 particles on the antiviral activity of ZnCl_2_, we first incubated EV-D68 virions with ZnCl_2_ for 2h before processing the virus attachment or entry assay (Figure 2D). Pre-incubation with ZnCl_2_ had no significant influence on EV-D68 entry (Figures 2E,F). Thus, our results suggested that zinc ions inhibited EV-D68 entry apparently due to the effect of ZnCl_2_ on the ability of cells to support virus replication rather than a direct effect on the virus itself.

### Zinc Ions Also Interfere With Release of EV-D68

**Figure 3:**
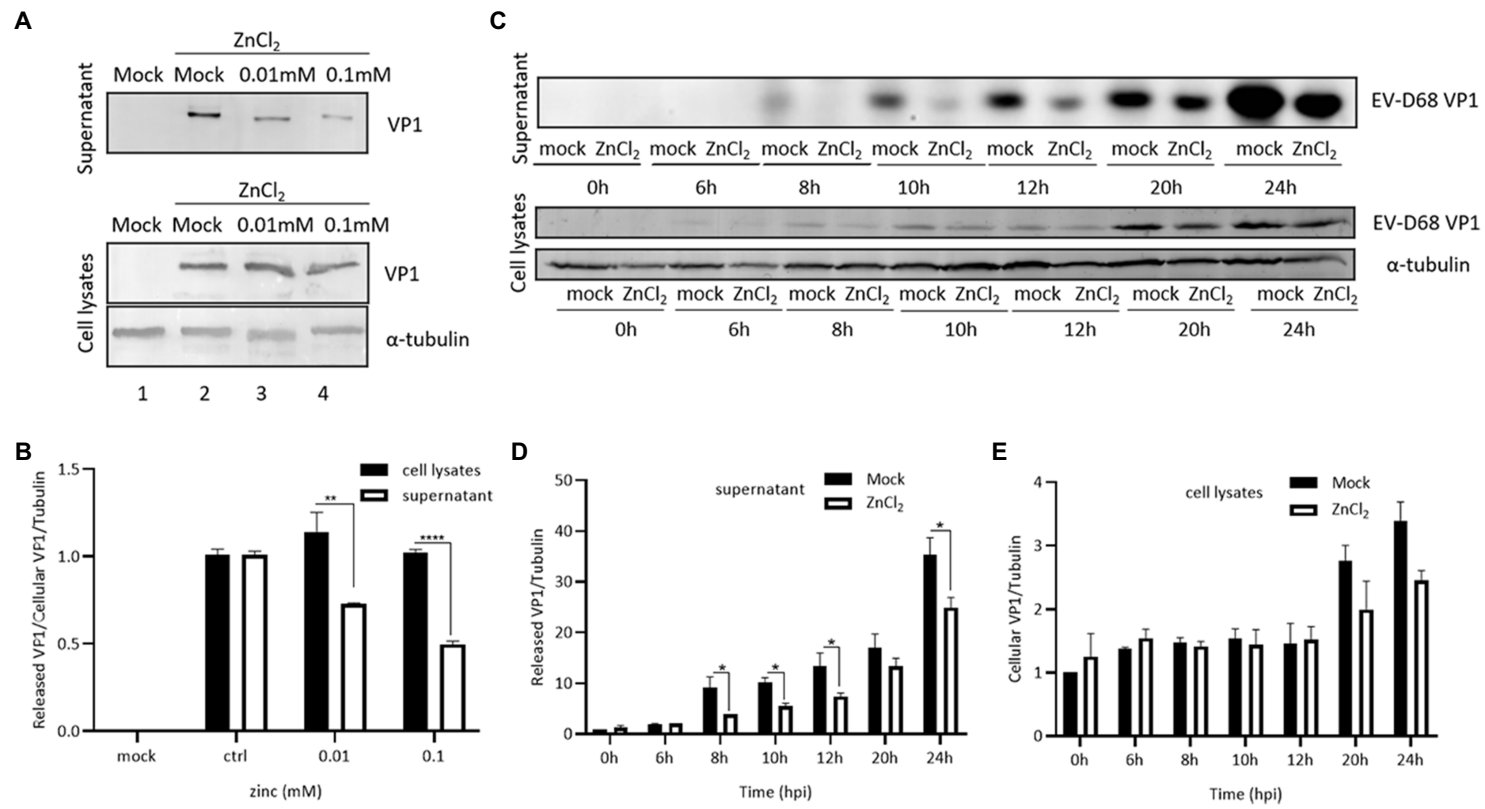
Zinc ions suppress release of EV-D68. **(A)** VP1 expression in supernatant and cell lysate at 48hpi. **(B)** The level of viral capsid protein expression from supernatant and cell lysate under same ZnCl_2_-treated concentration was compared with each other. **(C)** The changes of VP1 expression of the supernatant and cell lysates between the ZnCl_2_ treatment group and the control group at different times after infection. RD cells were treated with ZnCl_2_ at several specific time points, and the viral capsid protein expression level of supernatant **(D)** and cell lysates **(E)** was compared with that of the control group by Image J.

As our data showed that the inhibition of EV-D68 entry was milder than the suppression of EV-D68 replication by zinc ions, we suspected that zinc salts may also block viral replication at other steps in the life cycle of EV-D68. The levels of VP1 were used as indicators of the intracellular viral protein synthesis and viral particle release. RD cells were challenged with EV-D68 (MOI=0.1). After 48h, the cells and supernatant were harvested for the immunoblotting assay. ZnCl_2_ showed mildly effects on the expression of VP1 in the cell lysates, but potently decreased extracellular VP1 (Figures 3A,B). We then measured the changes in VP1 levels during infection in the presence or absence of ZnCl_2_ at different times after infection and found that ZnCl_2_ inhibited extracellular VP1 expression levels more potently than intracellular VP1. We also found that ZnCl_2_ exerted an inhibitory effect on the levels of extracellular VP1 after 8h, which was toward the end of the first life cycle of EV-D68 (Figures 3C–E). In summary, zinc ions seemed to exhibit dual antiviral activity against EV-D68 during the viral entry and release steps.

### Zinc Ions Inhibit Isolate 2014 US Circulating Strains of EV-D68

**Figure 4:**
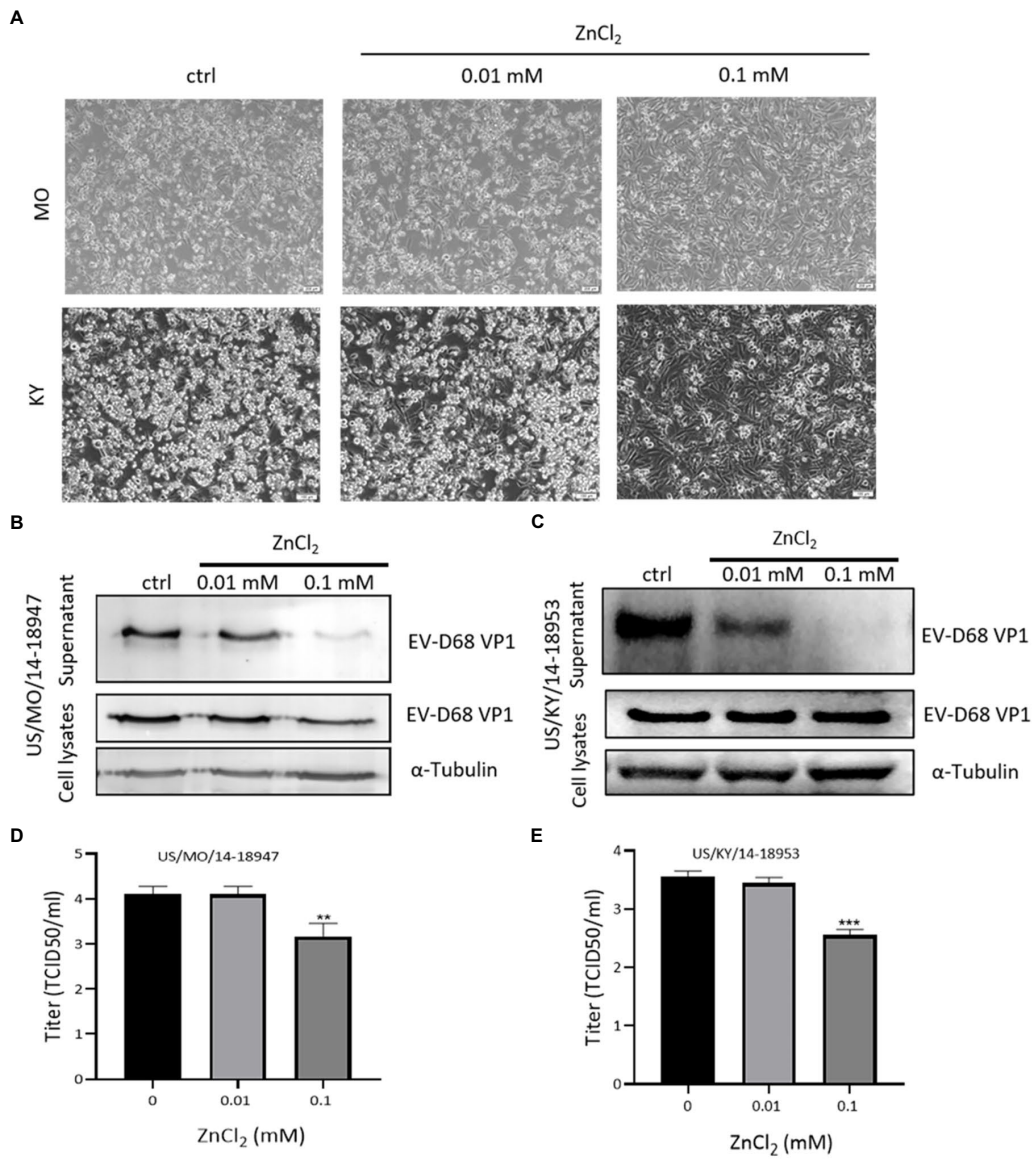
Zinc exerts inhibitory effects on US/MO/14-18947 and US/KY/14-18953 strains. **(A)** Cytopathic effects were observed at 48 hpi. **(B,C)** Immunoblotting showing VP1 expression at 48hpi. **(D,E)** Determination of progeny viral production. Supernatants were collected 48hpi, and viral titers were determined by standard plaque assay. Error bars indicate the SD (*p*<0.05; *p*<0.01; and *p*<0.001).

Recently, we and others demonstrated that the EV-D68 prototype virus and 2014 US circulating strains maintain different sensitivities for sialic acid-mediated viral entry (Wei et al., 2016). To evaluate the broad anti-EV-D68 activity of ZnCl_2_, we examined its antiviral activity against viral infection by primary EV-D68 isolates (US/MO/14–18947 [MO] and US/KY/14–18953 [KY]). The results showed that ZnCl_2_ treatment protected RD cells against both 2014 US circulating strains of EV-D68, as demonstrated by decreases in related CPEs (Figure 4A). The viral protein VP1 was also reduced by zinc ions (Figures 4B,C). Further, we also observed significantly higher suppression of VP1 levels in the supernatant than in the cell lysates, which further supported our conclusion that zinc ions maintained dual antiviral activities on virus entry and release. Furthermore, ZnCl_2_ treatment significantly decreased the virus titers of total virions of the MO and KY strains (Figures 4D,E), compared to the control group.

### Pyrrolidine Dithiocarbamate Enhances the Antiviral Activity of Zinc Ions Against EV-D68

**Figure 5:**
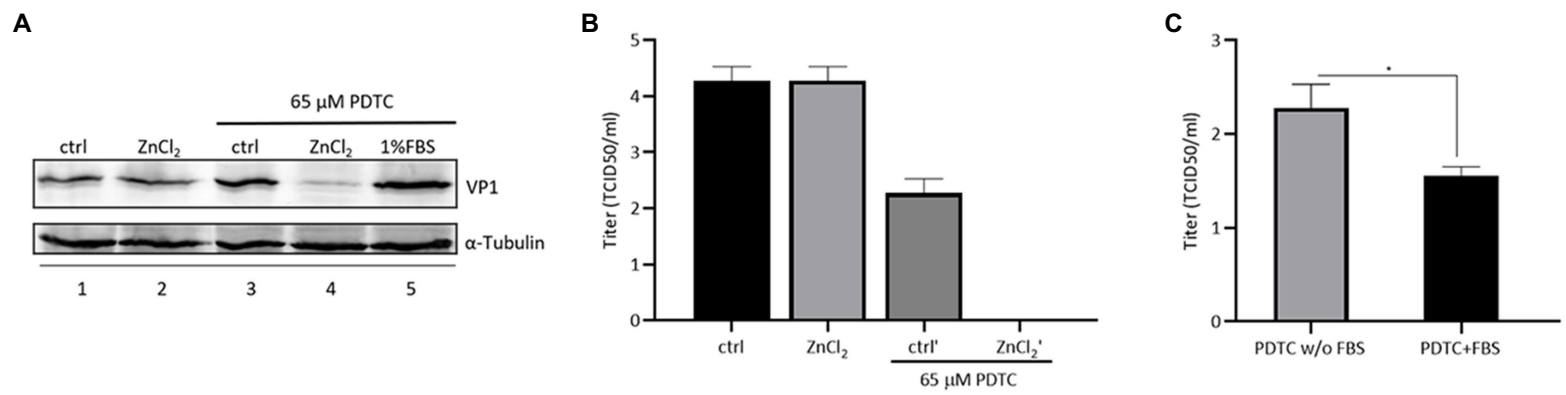
Combined treatment of zinc and pyrrolidine dithiocarbamate (PDTC) inhibits EV-D68 replication. **(A)** The combination of PDTC and zinc has an inhibitory effect on cellular VP1 expression. **(B)** The combination of PDTC and zinc decreases the amount of released virion of infected RD cells. **(C)** Infected cells were treated with PDTC with or without fetal bovine serum (FBS). The zinc ion in FBS helps PDTC to exert viral inhibitory effect.

Pyrrolidine dithiocarbamate (PDTC), a zinc ionophore, has been shown to restrict infection by different human viruses by facilitating the import of zinc ions into cells (Lanke et al., 2007; Qiu et al., 2013). We investigated whether PDTC could strengthen the anti-EV-D68 activity of zinc ions. We used a much lower concentration of ZnCl_2_ (0.0005mM) to treat RD cells, which showed no protective effect against EV-D68 infection (Figure 5). Meanwhile, a single treatment with PDTC at a concentration of 65μM also showed no influence on viral infection. Intracellular VP1 expression was suppressed only in the presence of both PDTC and ZnCl_2_, compared to the control group (Figure 5A). PDTC and ZnCl_2_ treatment significantly enhanced the resistance of RD cells to EV-D68 infection (Figure 5B). We noted the fold change of the titers of released virion is higher than that of intracellular VP1 expression in presence of PDTC treatment, which further supports our conclusion that EV-D68 inhibition by zinc influx is mainly targeting at virus release step. In addition, we confirmed that the zinc ions from FBS were associated with the antiviral activity of PDTC (Figure 5C). Hence, enhanced zinc ions influx could boost host cells against EV-D68 infection.

## Discussion

Enterovirus D68 and EV-A71 are the two major non-polio enteroviruses that circulate periodically around the world. Due to the current coronavirus disease of 2019 (COVID-19) pandemic, the number of reported EV-D68 cases has decreased in recent years. However, EV-D68 is still a non-negligible threat to public health due to its rapid spread and irreversible neurological toxicity. In our study, we demonstrated that zinc ions can suppress CPE and infectious virus generation by EV-D68 *in vitro*. Different zinc salts, namely zinc chloride, zinc sulfate, and zinc acetate, had identical effects on EV-D68 inhibition (Table 1). The inhibition was concentration-dependent and broad-spectrum for both the prototype and 2014 US circulating EV-D68 strains (Figure 4). Hence, our results support the notion that zinc supplementation could serve as a potential prophylactic and therapeutic treatment against EV-D68 infection.

Reportedly, zinc supplementation inhibits the replication of diverse viruses, such as herpes simplex virus (HSV), human immunodeficiency virus (HIV), and vaccinia virus (Korant et al., 1974; Katz and Margalith, 1981; Kumel et al., 1990; Baum et al., 2010). Zinc supplementation interferes with these viruses at different stages, including inhibition of viral uncoating, viral genome transcription, viral protein translation, and viral polyprotein processing (Fridlender et al., 1978; Kumel et al., 1990; Arens and Travis, 2000; Yuasa et al., 2006; Lanke et al., 2007; Liu and Kielian, 2012). Here, we found that zinc ions decreased the capacity of EV-D68 to attach to the targeted cells, which is the initial step for EV-D68 entry (Figure 2). Similar to this behavior, previous studies revealed that zinc ions can fit into the canyons on the surface of rhinovirus virions, thereby preventing the binding of viruses to the cell surface (Kumel et al., 1990). These results were consistent with previous cryo-electron microscopy structural studies that showed high similarity between the surface conformation of EV-D68 and rhinovirus (Liu et al., 2015b). Furthermore, we confirmed that the inhibition relied on the effect of ZnCl_2_ on the ability of cells to support virus replication rather than a direct effect on the virus itself.

In addition to the inhibition of EV-D68 entry, we also found that zinc treatment suppressed the total virion release from producing cells, by comparing the expression rates of VP1 in the cells and supernatant during viral replication (Figure 3). Hence, zinc ions potently blocked EV-D68 infection through the dual inhibition of virus entry and release. The detailed intracellular factors targeted by zinc ions, which are essential for its anti-EV-D68 activity, warrant further investigation. Nevertheless, the present study further enriched our understanding of the mechanisms of action of zinc salts against viral infections.

The physiological level of zinc in plasma is 10–20μM (Sugarman, 1983), and only about 10–20% of ingested zinc is absorbed. Increasing the sensitivity of host cells to EV-D68 inhibition by zinc salts could be helpful for the application of zinc salts in antiviral treatment. We found that the zinc ionophore PDTC, which facilitates the influx of zinc ions into cells, strongly amplified the antiviral activity of zinc. The concentration of ZnCl_2_ we used was much lower than the EC_50_, showing no significant effect on EV-D68 infection in single-treated cells, but potently suppressing virus infection in the presence of PDTC. These results showed that increasing the zinc influx may be a potential effective strategy for antiviral treatment.

It is important to further investigate EV-D68 infection and clinical outcomes in populations with zinc deficiency and in infants with zinc supplementation. Considering the safety of zinc usage, its easy availability, and the antiviral activity, the work described herein provides compelling evidence that zinc supplementation is an optimal therapeutic candidate for treating EV-D68 infections.

## Data Availability Statement

The original contributions presented in the study are included in the article/**Supplementary Material**; further inquiries can be directed to the corresponding authors.

## Author Contributions

WW, SL, and HG performed manuscript writing. WW, SL, HG, and XC carried out data analysis. WW contributed to study design. SL and HG performed data collection. All authors contributed to the article and approved the submitted version.

## Funding

This work was supported in part by funding from the National Natural Science Foundation of China (81772183 and 31800150), the National Science and Technology Major Project (2018ZX10731-101-001-016), the open project of Key Laboratory of Organ Regeneration and Transplantation, Ministry of Education (20202005), and the Department of Science and Technology of Jilin Province (Nos. 20190304033YY and 20180101127JC).

## Conflict of Interest

The authors declare that the research was conducted in the absence of any commercial or financial relationships that could be construed as a potential conflict of interest.

## Publisher’s Note

All claims expressed in this article are solely those of the authors and do not necessarily represent those of their affiliated organizations, or those of the publisher, the editors and the reviewers. Any product that may be evaluated in this article, or claim that may be made by its manufacturer, is not guaranteed or endorsed by the publisher.
