# Supplementary material for: Zinc Influx Restricts Enterovirus D68 Replication
Source: Front Microbiol. 2021 Oct 13;12:748546. doi: 10.3389/fmicb.2021.748546 (PMC8548656; doi:10.3389/fmicb.2021.748546)
Supplement: Supplementary file 1 [file Presentation_1.pdf]

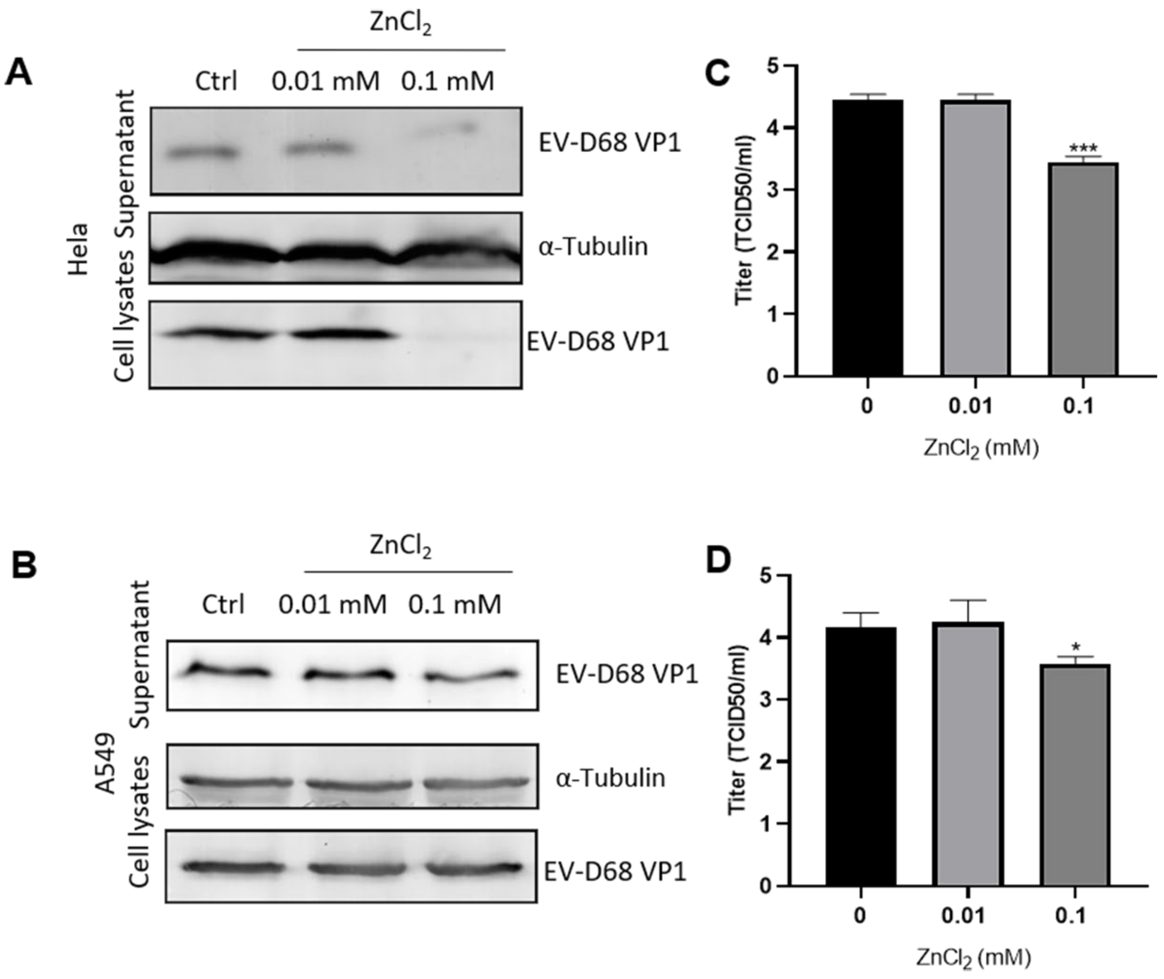


**Supplemental Figure 1. Zinc Chloride can protect A549 and HeLa cell lines against EVD68 infection.** (A) (B) Zinc inhibits the expression on EV-D68 viral protein in both A549 and Hela cells and released viral progeny. (C)(D) Zinc inhibits the replication of virions in infected A549 and Hela cells.
